# Reflections on tuberculosis diagnosis and treatment outcomes in Ghana

**DOI:** 10.1186/2049-3258-71-22

**Published:** 2013-08-23

**Authors:** Joshua Amo-Adjei, Kofi Awusabo-Asare

**Affiliations:** 1 Department of Population and Health, University of Cape Coast, Cape Coast, Ghana

**Keywords:** Tuberculosis control, Diagnosis, Treatment outcomes

## Abstract

**Background:**

Available evidence in Ghana shows the implementation of tuberculosis (TB) control activities efforts since the beginning of the 1900s. In spite of that, TB continues to be one of the common diseases in the country. In 1994, local and international policy windows opened for renewed strategies for the control of TB. This paper explores some of the approaches which have been in place since 1994 and their implications for treatment outcomes.

**Methods:**

The study combines quantitative and qualitative data. The quantitative data consist of treatment outcome from 1997–2010 and the qualitative data are derived from in-depth interviews with some staff of the TB control programme. Poisson regression and inductive coding were applied to the quantitative and qualitative data respectively.

**Results:**

Reported cure rates increased from 43.6% to 87.7% between 1997 and 2010. The data from the in-depth interviews (IDIs) suggested that improvements in diagnosis, community TB care, stigma reduction among community and health workers towards TB patients, the public-private partnership, and the enablers’ package contributed to the improved better treatment outcomes, particularly from 2008.

**Conclusions:**

Lessons learnt include the achievement of objectives with varying strategies and stakeholder interventions. Further studies would be needed to quantify the contributions of the various interventions to help determine those that are cost effective as well as efficient and effective for TB control.

## Background

As part of the measures to reduce, if not eliminate, the adverse effects of TB, a number of interventions and strategies have been applied, from the individual, societal, national and international levels since the disease was first identified in the 1800s [1]. At the individual level, various strategies have been adopted, for instance “purging, bed rest, horseback riding, the mountains, the seashore, cod-liver oil, castor oil, phrenic nerve interruption, thoracoplasty, pneumothorax, lucite ball, air in the chest, air in the abdomen … the list of attempted remedies from the Greeks to the moderns (sic) seems nearly infinite” ([2], p.11).

The provision of sanatoria was also seen as one of the effective broad scale approaches to controlling TB infection and cure [3]. Others were public health interventions such as mass screening and vaccinations, which have been claimed to contribute to reducing TB infections [4,5] and at the societal level, the implementation of poverty reduction strategies in some parts of the world, especially the developed countries [6,7].

The body of knowledge on factors contributing to the decline in the TB infections and death have been diverse and sometimes saddled with controversies, especially those, which deal with interventions prior to the development of chemotherapy. For instance, McKeown [8] has emphasised the contribution of improved nutrition to the decline of TB, while Szreter [9] has argued around the impacts of broad social interventions such as legislations against overcrowding at residential and industrial areas as factors which accounted for the decline of TB in some parts of Europe.

Recent approaches, often socio-medical in nature have also accounted for improved diagnosis and treatment of TB. For instance, the reduction in treatment duration from 18 months to six months [10] is considered to have led to improvement in compliance to treatment and subsequently low defaulter rates. Similarly, other measures such as fixed-dose combination [11], community treatment care [12], enablers package [13], standardised treatment [14], the establishment of Green Light Committee (GLC) and Global Drug Facility to facilitate reliable drug supply [15], practical approach to lung disease (PAL) [16], and public private partnership [17] have been introduced with the objective of reducing the burden of TB on individuals and communities.

In Ghana, attempts to deal with TB dates back to the early 1900s. However, those attempts were sporadic and uncoordinated. Formalised institution for TB control came into being in 1959 with the establishment of the Ghana TB Services [18]. However, towards the end of the 1960s through to the early 1990s, activities geared towards the control of the disease declined substantially [19]. The HIV/AIDS epidemic, which became prominent around the world in the early 1990s heightened and increased re-emergence of TB led to renewed interest in TB control at the international level, also led to change in strategies and policies in the country.

For instance, with the increasing burden of TB, partly fuelled by HIV/AIDS epidemic, the WHO, in 1993, declared TB a global emergency, calling for new efforts and commitments for control of the disease. Country health departments/ministries were called upon to formalise and establish national response programmes. In response to such calls, Ghana established a new TB control programme in 1994.

Despite the fact that Ghana is not one of the high burden TB countries in Africa, it nevertheless considers TB as an important health challenge. Together with HIV, they account for about 7% of all deaths, the second after malaria [20]. It is also estimated that less than half of TB in the country are notified. For example, in 2010, only 15,145 of the 47,632 projected cases were diagnosed [20].

This paper tracks TB treatment outcomes in the country and explores possible social and biomedical interventions which are perceived to have contributed to the fight against the disease in Ghana. The aim is to identify the social and technical strategies which have been implemented since 1994, and their implications for TB diagnosis and treatment outcomes.

## Methods

This paper was extracted from a larger study which investigated policy environment for TB control in Ghana. Among the issues examined in the larger study were historical perspectives on the disease, public-private partnership, integration of TB and HIV control programmes, and obstacles and opportunities for TB control interventions.

This study combined quantitative and qualitative methods, leading to a mixed method approach [21]. The quantitative data are treatment outcomes, available from 1997 to 2010 from the National Tuberculosis Control Programme (NTP). The data were collected through passive surveillance and are disaggregated by region and the two tertiary hospitals in the country, the Korle-Bu Teaching Hospital (KABTH) and the Komfo Anokye Teaching Hospital (KATH). The treatment outcome information available in the data set is the number of people cured, completed treatment, died, treatment failure, and defaulters. A TB case is categorized as cured when a patient completes all prescribed doses and is documented to have recorded two or more consecutive negative cultures after six months of starting treatment. Treatment completion occurs when all prescribed doses are adhered to but lacking bacteriologic proof of cure because a patient is unable to produce sputum. Death occurs if during the treatment period, the patient dies with TB as the major cause of death, or death arising from toxicity due to anti-TB medications after receiving at least one week of anti-TB medications. Treatment is categorized as a ‘failure’ if there is presence of positive culture for *M. tuberculosis* after four months of treatment. Treatment default is when treatment is interrupted for two or more consecutive months after initiation of treatment. Because the treatment outcome data were captured as count data, Poisson regression was used to analyse the treatment outcomes data. This was chosen because, first, the data did not contain any zeros to warrant the application of Zero-inflated and also, the data did not show evidence of dispersion to permit the use of Negative binomial regression. Simple linear regression will also not be applicable in this case because the data was not collected through random sampling. The results are presented in incident rate ratio (IRR).

The qualitative data was collected from national, regional, district and health facility TB coordinators and service providers. At the national level, three senior programme officers were interviewed, two of whom had been with the programme since 1994 and thus were able to provide detailed information about the programme. In addition to the national officers, four regional TB coordinators were selected based on the recorded TB case notifications in these regions. In each of the regions selected, the district with the highest notification rate of TB was chosen and the district coordinators (4) included in the study. In each district, three facilities (2 public and 1 private), and TB coordinators were interviewed. Finally, one respondent who works with a TB-related international NGO was also interviewed, resulting in overall sample size of 24 respondents. (The 24 respondents constitute a sub-sample of a total of 31 respondents in the larger study). Semi-structured interview guide used covered issues in TB control such as political commitment, public-private partnership, integration of TB and HIV services and sustainability current interventions. All the interviews were tape-recorded, transcribed and analysed manually. The qualitative data analysis plan followed an inductive approach as reported elsewhere [22]. The University of Cape Coast Institutional Review Board reviewed, and gave ethical approval for the study.

## Results

Results of the study are reported in two sections: first is analysis of TB treatment outcomes based on the quantitative data and the second deals with perceived factors which may have contributed to the observed results using the qualitative data.

### TB treatment outcomes: 1997 – 2010

**Figure 1:**
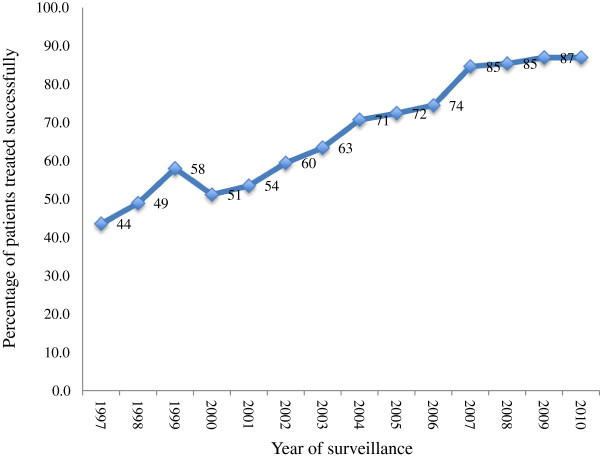
Proportion of TB patients cured successfully between 1997–2010 in Ghana.

Regardless of the number of reported TB cases in a setting, key treatment outcomes are expected to improve in line with WHO treatment outcomes benchmarks. Currently, international benchmark for assessing countries’ performance in respect of cure rates is 85%. Figure 1 shows the proportion of patients who were cured between 1997 and 2010 ranged from 44% in 1997 to 87% in 2010. Cure rates for the first decade of TB surveillance were below the WHO thresholds of 85 per cent but improvements are noted from 2008 onwards.

While there was increase in the cure rates, the proportion dying and experiencing treatment failure or default declined. The Poisson regression outputs confirming these are shown in Table 1. The overall likelihood of cure was significantly highest in the Greater Accra (IRR = 1.359; *p* < 0.001) while the least incident rate ratio of cure was reported at the Komfo-Anokye Teaching Hospital [KATH] (IRR = 0.00991; *p* < 0.001). [The Western Region was used as the reference category because it reported one of the highest TB rates in the country]. Completed treatment was more likely in the Western compared to the others. TB deaths were more likely in the Eastern Region, approximately 55% compared to the Western Region. The rate of treatment failure was significantly lower in the Upper West Region.

**Table 1:** Tuberculosis treatment outcomes by region and surveillance year in Ghana, 1997-2010

**Observations**	**Cure**		**Completed**		**Died**		**Failure**		**Default**	
	**IRR**	**95% CI**	**IRR**	**95% CI**	**IRR**	**95% CI**	**IRR**	**95% CI**	**IRR**	**95% CI**
Central^1^	0.990	[0.960,1.020]	0.438^***^	[0.399,0.480]	1.376^***^	[1.256,1.508]	0.842^*^	[0.723,0.979]	1.714^***^	[1.618,1.816]
Greater Accra	1.359^***^	[1.321,1.399]	0.393^***^	[0.357,0.432]	1.061	[0.962,1.169]	0.689^***^	[0.586,0.808]	0.819^***^	[0.765,0.877]
Volta	0.828^***^	[0.802,0.855]	0.494^***^	[0.452,0.540]	0.903^*^	[0.816,0.999]	0.350^***^	[0.286,0.428]	0.417^***^	[0.383,0.454]
Eastern	0.970^*^	[0.940,1.000]	0.631^***^	[0.581,0.685]	1.545^***^	[1.413,1.689]	0.702^***^	[0.599,0.824]	1.002	[0.939,1.069]
Ashanti	1.215^***^	[1.180,1.251]	0.939	[0.873,1.011]	1.315^***^	[1.199,1.442]	0.391^***^	[0.322,0.474]	0.526^***^	[0.486,0.569]
Brong-Ahafo	0.470^***^	[0.452,0.488]	0.416^***^	[0.378,0.457]	1.011	[0.917,1.116]	0.571^***^	[0.482,0.677]	0.351^***^	[0.321,0.384]
Northern	0.230^***^	[0.219,0.242]	0.247^***^	[0.219,0.278]	0.334^***^	[0.290,0.386]	0.165^***^	[0.124,0.220]	0.162^***^	[0.142,0.184]
Upper East	0.243^***^	[0.232,0.256]	0.0946^***^	[0.0795,0.113]	0.579^***^	[0.516,0.649]	0.139^***^	[0.104,0.187]	0.114^***^	[0.0985,0.131]
Upper West	0.0973^***^	[0.0905,0.105]	0.168^***^	[0.147,0.192]	0.267^***^	[0.230,0.311]	0.0929^***^	[0.0654,0.13]	0.0802^***^	[0.0677,0.0949]
KATH	0.00991^***^	[0.0079,0.0123]	0.296^***^	[0.266,0.330]	0.127^***^	[0.104,0.157]	1.049	[0.909,1.211]	0.0681^***^	[0.0568,0.0817]
KBTH	0.0145^***^	[0.0121,0.0174]	0.287^***^	[0.257,0.319]	0.209^***^	[0.177,0.247]	0.926	[0.799,1.074]	0.120^***^	[0.104,0.138]
1998^2^	1.287^***^	[1.222,1.356]	0.861^*^	[0.765,0.969]	1.046	[0.933,1.173]	0.798^**^	[0.675,0.945]	0.886^**^	[0.822,0.955]
1999	1.234^***^	[1.171,1.301]	0.728^***^	[0.644,0.824]	0.872^*^	[0.773,0.983]	0.921	[0.783,1.083]	0.818^***^	[0.758,0.883]
2000	1.181^***^	[1.120,1.245]	0.679^***^	[0.599,0.770]	0.788^***^	[0.697,0.891]	0.743^***^	[0.626,0.883]	0.671^***^	[0.619,0.728]
2001	1.327^***^	[1.260,1.397]	1.113	[0.996,1.243]	0.803^***^	[0.711,0.908]	0.766^**^	[0.646,0.908]	0.867^***^	[0.804,0.934]
2002	1.525^***^	[1.450,1.604]	0.786^***^	[0.696,0.887]	0.937	[0.833,1.053]	0.663^***^	[0.555,0.791]	0.731^***^	[0.676,0.791]
2003	1.695^***^	[1.613,1.781]	0.866^*^	[0.770,0.974]	1.024	[0.913,1.149]	0.498^***^	[0.410,0.604]	0.711^***^	[0.657,0.770]
2004	1.724^***^	[1.641,1.811]	0.971	[0.866,1.088]	0.942	[0.837,1.059]	0.495^***^	[0.407,0.601]	0.647^***^	[0.596,0.702]
2005	1.921^***^	[1.830,2.016]	0.751^***^	[0.665,0.849]	1.092	[0.975,1.223]	0.478^***^	[0.393,0.582]	0.579^***^	[0.532,0.630]
2006	2.052^***^	[1.956,2.152]	0.822^**^	[0.729,0.926]	1.092	[0.975,1.223]	0.501^***^	[0.413,0.608]	0.326^***^	[0.294,0.361]
2007	2.145^***^	[2.046,2.250]	0.930	[0.828,1.044]	0.981	[0.873,1.102]	0.352^***^	[0.283,0.439]	0.153^***^	[0.133,0.176]
2008	2.324^***^	[2.218,2.436]	1.062	[0.950,1.188]	0.885^*^	[0.786,0.997]	0.346^***^	[0.277,0.431]	0.123^***^	[0.106,0.144]
2009	2.496^***^	[2.382,2.615]	1.139^*^	[1.020,1.272]	0.846^**^	[0.750,0.955]	0.281^***^	[0.222,0.357]	0.152^***^	[0.132,0.175]
2010	2.206^***^	[2.104,2.313]	1.285^***^	[1.154,1.430]	0.877^*^	[0.778,0.988]	0.339^***^	[0.272,0.424]	0.163^***^	[0.142,0.186]
Constant	343.2^***^	[328.3,358.7]	113.1^***^	[103.0,124.1]	60.15^***^	[54.13,66.84]	44.73^***^	[38.61,51.82]	232.7^***^	[217.7,248.7]
Log likelihood	−2172.6		−1052.5		−910.9		−735.6		−1435.3	
Chi-squared	47983.4		2994.5		3028.6		1306.1		13179.8	
*N*	167		167		167		167		167	

*p* < 0.05, ^**^p < 0.01, ^***^p < 0.001.

Reference groups are as follows: ^1^Western; ^2^1997.

Treatment outcomes varied by year of surveillance: the rate ratio of cure and treatment failure was lowest in 2009 and treatment completion was higher in 2010 than any of the earlier periods. The rate of TB death was significantly more likely in 1998, 2003, 2005 and 2006. Default in treatment was less likely in 2008. In respect of overall default, outstanding results are observed after 2006 (Table 1).

When decomposed by region and year (data not shown) and we found that in 2009, the likelihood of cure was significantly more likely in the Northern Region in 2009 (using 1997 as the base year) while the least cure rates were reported at the Korle-Bu Teaching Hospital for the years 2006, 2008 and 2009.

### Qualitative evidence

#### Background of respondents and themes

The number of years of experience of the respondents varied from one level of service to another. Overall, the respondents had been in service for durations ranging from 2–15 years. The national officers and few regional personnel had been involved in TB control longer than the others, especially compared to those at the facilities. At the facility levels, there were indications of attrition due to regular postings. The conversation with the respondents revolved around factors contributing to the trend in the number of TB cases identified and the corresponding treatment outcomes. Depending on experience and the duration respondent had been involved in TB control; views on determinants of the treatment outcomes were diverse. The qualitative data collection was partly used to document perceptions on the management and outcomes of the various strategies which had been adopted since 1994. The following themes were extracted from the interviews: improvement in case management; improved diagnosis; the introduction of community TB care and enablers’ package, stigmatisation of TB patients, interest of health personnel in TB management, and the involvement of private sector in TB control.

#### Improved TB case management

Respondents were of the view that improvement had occurred in case management and this was reflected in treatment outcomes, for example, cure and default rates. The respondents generally believed that more patients were now complying with treatment regimens than before. A low defaulter rate is beneficial to the individual patient as well as the larger health system as defaulting is associated with drug resistant bacteria [23]. The following excerpt from a respondent demonstrates how declining default rates is viewed:

What I consider to be a major outcome of the new strategy is the reduction in defaulter rates. I learnt that defaulter rates in the region were around 20% in the past but presently; it has reduced to around 4-5% (Coordinator, Region 1).

The most prominent reason respondents gave for the decline in default rates was the introduction of a fixed dose combination therapy, which has reduced the treatment period to standardised six months, which hitherto, ranged from eight to 18 months. Fixed-dose combination (FDC) is a condensation of first-line drugs: ethanbutol, isoniazid, rifampicin and pyrazinamide into one dosage [24]. FDC can reduce the risk of incorrect dosage, simplify drug procurement and improves adherence [11]. Thus, it was noted that:

… The result of the cutting down on treatment period from eight to six months for non-resistant TB has improved completion rate and subsequently, cure rates. Previously, the long duration for treatment led to more defaulters (Region 2 Coordinator).

Improvements in technology which led to decline in the duration of administration of the drugs appears to have translated into adherence to drug regimens.

#### Improvement in diagnosis

Improvement in the diagnosis of suspected TB patients was also considered to be one of the important components of the TB control programme. To them, early and accurate diagnosis of suspected cases made it possible for treatment to start early. Because a single TB patient could transmit the disease to about 10 persons per annum, the identification of even one case at its early stage was important to reducing new infections. A respondent had this to say about improved diagnosis:

We have ensured that the quality of sputum produced for diagnosis is appreciable because confirmation of cases is difficult or impossible without laboratory confirmation. In line with this, every patient is given two containers for sputum specimen – one for early morning specimen and other for on-the-spot specimen. We observed from this initiative that the quality of sputum improved … after implementing coaching of suspects, there were slight changes, about 2% in quality of sputum and we expect this to continue improving … quality sputum allows us to have proper bacilli formation through culturing (Region I, Laboratory Focal Person).

It has been reported that poor diagnosis was a major hindrance to TB control because imprecise diagnosis could result in false negative results. The provision of information on reasons for the collection of sputum, how to produce the sputum; information on characteristics of quality sputum have had impacts on the quality of sputum, leading to positive sputum microscopy analysis [25].

#### Community TB treatment care

One of the elements of the new approach to TB treatment is the participation of community members. The community treatment care concept recognises social capital and connectedness as instrumental to effective TB control. Under this intervention, individual TB patients are assigned a “significant other” to motivate and supervise patients to take his/her drugs. Health workers in collaboration with patients do the selection of treatment supporters. Some respondents were of the view that community involvement had played a very important role in improving treatment completion and helped to reduce defaulter rates. As noted by one of the respondents:

Community involvement has helped to reduce defaulter rates because some selected individuals within patients’ immediate environment help to supervise treatment without necessarily travelling long distances for treatment (Region 2 Coordinator).

To some of the respondents, the use of treatment supporters who could be family members, religious leaders, health workers or other gatekeepers who the patient trusts and feel comfortable dealing with might have contributed to the improved adherence to treatment.

#### Decline in stigma and response of health personnel to TB management

The available evidence suggests that stigma of infected people and the affected poses some amount of threat to effective TB control [26]. Stigma may discourage infected persons from seeking treatment early. One of the strategies was to engage communities in stigma reduction programmes. Even among nurses, it was considered a punishment to be transferred to the chest clinic but this is changing [25]. A senior national officer noted that the changes in health workers attitudes, in particular are contributing positively to stigma reduction and the overall management of the disease. He noted:

Now even the cadres of health worker coming in are people who have opted to work with the TB control programme. Previously, it was considered a punishment to be posted to chest clinic. Personnel who were considered stubborn, indiscipline or rebels in the system were sent to DOTS centres – it was like Siberia. But that is changing. There is increasing commitment from health workers to be involved in TB control and this is helping a lot (National Officer, A).

Another respondent at the regional level also expressed satisfaction with the changing attitude of health workers towards TB control.

It was a disease people did not want to get closer to; if you were in the chest clinic, even your colleagues were not willing to come close to you. But now, people feel comfortable working with TB patients. Although resources in TB may be a factor, the important thing is that some health workers have gone through training programmes to work with TB patients. Through these training programmes, we have been able to demystify people’s perception that TB is a dangerous (District Coordinator, Region 2).

Funded disease control programmes such as TB, HIV/AIDS and Malaria tend to attract personnel, apparently, because of the financial resources associated with those programmes. Although the respondents were aware of the resources involved in TB control, their interests could not be attributed to the resources. Another reason respondents cited, as accounting for the changes in the attitude of health workers was the positive treatment outcomes being recorded – the perception that staff at the facilities are seeing results from their labours has given some of them further impetus to continue. As observed by one respondent:

People see a clear vision for TB control, knowledge has improved, there is greater understanding, the disease has been demystified, logistics needed for control are available – workers don’t have to shout and shout for resources … health workers are beginning to see results, which translate into job satisfaction. If you treat somebody and notice the patient is getting healed, you become content (National Officer A).

The claims by some of the senior management of the NTP at the national and regional levels were collaborated by personnel who were directly dealing with patients. Generally, the respondents at the health facilities confirmed the views expressed by the top management as many of them indicated that they willingly accepted postings to DOTS centres. Their argument was that it was professionally ethical for a health worker to accept to work on categories of patients without prejudice and discrimination.

I accepted to come here because I have been trained to provide health care to all those who need it, regardless of their condition. There was no reason for me to refuse posting to the DOTS centre (DOTS Centre Nurse, Region 2, Public Hospital).

#### The enablers’ package

An enablers’ package include a wide range of services such as travel vouchers, reimbursement, cash payments, toiletries, clothing, cell phone minutes, food during DOT visits, vouchers, periodic food packages; social welfare payments during treatment; income generation project; salary payments, legal services, housing or housing subsidies, personalised incentives or “bait for fishermen” (providing other payments to community or patients who bring suspects for treatment) [27]. Cash payments or travel vouchers (both staff and clients), food supplements/packages and social welfare payments are some of the popular enablers’ support in Ghana. One respondent described how she felt about the enablers’ package in the following extract:

Among the poorest of clients, they were given transportation, beverages and then home support visit … The enablers’ package was an encouragement factor; people came because their relations had told them to do so due to enablers’. This pushed people with chest/lung ailments to come on their own accord due to that small token.

The enablers package also covered travel vouchers for health workers and this, according some respondents, it facilitated home verification, etc.

#### Public-private partnership

Public-private partnership (PPP) for TB control is important of its effects on bridging access gaps [28]. A section of the respondents felt that the PPP has made modest contributions to TB control in Ghana, although there is no disaggregated data on TB treatment outcome by type of facility [19]. In the view of the respondents, the PPP approach has the potential of improving access. A respondent in one of the regions where the PPP was piloted had this to say:

It worked well in the few places where it was first started. We are able to manage many patients who could have been lost to follow-up, given that there are only five public hospitals in this metropolis providing DOTS services. At one point, we were recording no defaulters because almost every available private facility was involved in DOTS, even maternity homes (Coordinator, Region 2).

## Discussion

This paper has offered some insights about TB treatment outcomes in Ghana between 1997 and 2010. Coupled with that, attempts have been made to explore the views of key personnel involved in TB control about the possible patient and health system-based factors which have played important roles in achieving the current treatment outcomes. In spite of the modest contributions of this study to our understanding of TB treatment outcomes and the possible factors shaping these outcomes, we are limited to make attributions. However, the findings shed light on possible interventions and strategies which could be studied further to ascertain their measureable impacts on TB control in Ghana. The qualitative evidence is also not representative of the whole NTP establishment.

Importantly, our results point to incremental improvements in five specific TB treatment outcomes: the rate of cure, completed treatment, treatment failure, default and death from TB. The timing (year) and places (region) where the best results were achieved on each of the treatment outcomes varied significantly. However, in 2009, impressive results were achieved on all outcome measures. Follow-up discussions at the headquarters of the NTP revealed that in 2009, there was high participation of TB related-NGOs in advocacy on the need for testing, early treatment seeking and other equally important components of TB management.

Of comment, another relevant finding, is the high likelihood of TB deaths in the Eastern Region. The Eastern Region of Ghana has consistently recorded the highest burden of HIV [29]. HIV infection does not only reactivate latent TB infection but also accelerates new infections and re–infections, with a lifetime risk of TB occurring among HIV patients ranging from 10–20% [30]. Without timely initiation of joint TB and HIV treatment, the likelihood of TB mortality could be significantly higher. The HIV situation in the Eastern Region may therefore be a contributory factor the high likelihood of TB mortality in the region.

The results further point to poor TB treatment outcomes at the two tertiary hospitals, particularly, the Korle-Bu Teaching Hospital. Whereas a number of factors may be contributing to the outcomes at the tertiary hospitals, few are hypothesised. First, as some respondents recounted, community treatment care and regular follow-ups on patients have been critical to treatment outcomes. However, because these hospitals are referral and also located in cosmopolitan areas, follow-up on patients may not be effective and efficient as it possibly happens at the lower levels of health delivery. Further studies that compare the quality of care at tertiary hospitals on the one hand and lower level hospitals/clinics on the other hand may help us get better understanding of these issues.

Adherence to TB treatment is an important consideration in TB control programs because incomplete treatment can lead to prolonged infection, drug resistance, relapse, and death [13]. The enablers’ package, an intervention, which seeks to motivate patients and health workers, highlighted as a factor in improving treatment outcomes. This finding re-enforces the relevance of the package, especially the fact that it is health workers who are highlighting on its importance. Much of the evidence on the usefulness of the enablers’ package has come from patient beneficiaries.

It was also noticed that the increasing interest of health workers in TB control efforts is not arising from the amount of financial resources available to the TB programme, although an earlier study on barriers to the control of TB had revealed monetary incentives as one of the barriers [22]. According to this study, the financial resources in the TB control programme had unfortunately led to “personalisation” of activities by some TB coordinators. Financial motivations for health personnel on programmed diseases can be counter-productive [31]. Some part of the existing evidence suggests that as long as there are funds, such diseases are attractive to many personnel. For instance, In Burkina Faso, it was found that the interest of health workers in HIV/AIDS was far more than the interest in reproductive health [31].

The findings also suggest that a reverse causality between treatment outcomes and the interest of health workers in TB control. Some respondents perceived that with the rising positive outcomes in treatment, health workers are becoming more committed to TB management and this in turn served as the motivation to work harder. In their review of motivations of health workers, Dolea and Adams [32] found that non-financial incentives such as better treatment results served as intrinsic motivation to health workers.

In keeping with earlier studies [17,27,33,34], this study also highlights the need to up-scale the involvement of the private sector in TB control. The study participants generally indicated that the PPP approach has made positive contribution to TB control. Despite the importance of the private sector in TB control, sub-standardised DOTS treatment has been reported [35]. The inclusion of private sector in TB control therefore requires constant monitoring and supervision in order to assure and maintain standardised treatment.

## Conclusions

Overall, the findings can be summarised into two broad areas: the perceived contributions of social and biomedical interventions. Social interventions included the enablers’ package, community participation in treatment and the public-private partnership. The contributions of biomedical interventions were linked to better case management through the use of FDC and improvement in diagnosis and confirmation of cases. It appears from the findings that an effective combination of social and biomedical interventions may be able to accelerate the global efforts towards significant improvements in TB control. While no attempts were made to match the specific interventions with any particular outcomes due to the exploratory nature of the study, it can be concluded that the interventions mentioned by the respondents (albeit not exhaustive) were perceived to have contributed to better treatment outcomes of TB in Ghana. The interventions noted and other novel ones should be pursued by the policy makers to help achieve the goals set for TB.

## Competing interests

The authors declare they have no competing interests.

## Authors’ contributions

JAA wrote the first draft of the manuscript. KAA revised it critically and has given final approval of the version to be published. Both authors read and approved the final manuscript.

## Authors’ information

JAA is completing PhD in Population and Health, University of Cape Coast with current interest on tuberculosis policies. KAA is a professor of Population Health, University of Cape Coast.
